# Determinants of low birth weight and its effect on childhood health and nutritional outcomes in Bangladesh

**DOI:** 10.1186/s41043-024-00565-9

**Published:** 2024-05-13

**Authors:** Md. Zahidul Islam, Mohammad Rocky Khan Chowdhury, Manzur Kader, Baki Billah, Md. Shariful Islam, Mamunur Rashid

**Affiliations:** 1 Department of Public Health, First Capital University of Bangladesh, Chuadanga, Bangladesh; 2 Department of Epidemiology and Preventive Medicine, School of Public Health and Preventive Medicine, Monash University, Melbourne, Australia; 3 Department of Medical Science, School of Health and Welfare, Dalarna University, Falun, Sweden; 4 Department of Public Health and Sports Sciences, University of Gävle, Gävle, Sweden

**Keywords:** Low birth weight, Child health, Undernutrition, Environmental factors, Bangladesh

## Abstract

**Background:**

The high incidence of low birth weight (LBW) is associated with an increased risk of infant mortality, adverse pregnancy outcomes for mothers, and a decline in overall health and well-being. The current study aimed to identify the various determinants of LBW and its effect on adverse health and nutritional outcomes of children aged 0–23 months in Bangladesh.

**Methods:**

Bangladesh Demography and Health Survey (BDHS) 2017-18 data was used. A chi-square test and multivariable logistic regression analysis were used to find out the associations between independent variables and outcomes (e.g., LBW, child illness and undernutrition).

**Results:**

The overall prevalence of LBW among was 16.3%. Mother with no formal education (AOR = 2.64, 95% CI = 0.55–3.30, *p* = 0.01), female child (AOR = 1.31, 95% CI = 1.04–1.65, *p* = 0.023); and poorest economic status (AOR = 1.69, 95% CI = 1.13–2.51, *p* = 0.010), were identified significant determinants of LBW. Of home environment and hygiene factors, unimproved toilet facilities (AOR = 1.38, 95% CI = 1.03–1.84, *p* = 0.030) had a significant effect on LBW. In addition, children born with LBW were more likely to suffer fever (AOR = 1.26, 95% CI = 1.05–1.60, *p* = 0.050), stunting (AOR = 2.42, 95% CI = 1.86–3.15, p = < 0.001), wasting (AOR = 1.47, 95% CI = 1.02–2.25 *p* = 0.049), and underweight (AOR = 3.19, 95% CI = 2.40–4.23, p = < 0.001).

**Conclusion:**

One out of five children was LBW in Bangladesh. Maternal education, sex of child, wealth index, and toilet facilities had significant effects on LBW. In addition, LWB contributed to children’s poor health and nutritional outcomes. Enhancing maternal pregnancy, and child health outcomes necessitates policies addressing poverty, gender inequality, and social disparities. Key strategies include promoting regular prenatal care, early medical intervention, reproductive health education, and safe hygiene practices. To combat the negative impacts of LBW, a comprehensive strategy is vital, encompassing exclusive breastfeeding, nutritional support, growth monitoring, accessible healthcare, and caregiver education.

**Supplementary Information:**

The online version contains supplementary material available at 10.1186/s41043-024-00565-9.

## Background

Low birth weight (LBW) of children poses a serious public health problem in low- and middle-income countries [1]. Early childhood is a critical window for children’s physical and mental development and LBW contributes as a leading cause of illness and death among children during this period [2]. Two physiological conditions among others, Intrauterine Growth Restriction and/or preterm birth during pregnancy can basically lead to children’s born with LBW [3]. LBW is responsible for 60–80% of the total mortality in children under one month of age and one-third of total deaths among children aged less than one year [4, 5]. Further, the likelihood of infant mortality is 40 times higher among LBW children compared to normal children [4]. Apart from mortality, it hinders normal growth and raises the risk of developing chronic illnesses, such as ischemic heart disease, diabetes, dementia, osteoarthritis, stroke, and hypertension, later in life [6–8]. LBW also increases the chances of developing behavioral and psychological disorders, as well as sensory and learning disabilities [9, 10]. Furthermore, compared to normal infants, those who are born with LBW are at a greater risk of experiencing prolonged and intense infections, such as diarrhea and acute respiratory infection (ARI), which are the leading causes of child mortality [6].

Around 30 million infants worldwide, accounting for 23.4% of all newborns annually, are born underweight [7]. This condition can result in numerous immediate and extended health and nutritional complications. The prevalence of LBW is considerably higher in low- and middle-income countries, with the estimation of South Asia (28%) and Sub-Saharan Africa (13%) was being most affected regions. This highlights the existing health inequalities between different parts of the world [11, 12]. The rate of LBW in Bangladesh dropped to 14.5% in 2022, showing a significant decline from the 20% recorded in 2012 [2, 9].

Despite substantial efforts were made to uncover the etiology of LBW in several research, yet the etiology of LBW is not well understood [13, 14]. LBW was determined by the complex interplay of several factors including biological (such as, premature birth, intrauterine growth restriction, genetic factors, etc.) maternal (age, body mass index, education, occupation, maternal mental stress, maternal weight gain during pregnancy, mother’s access to prenatal care, diet during pregnancy and others), environmental (natural disaster, type of toilet facilities, type of drinking water, used solid waste for cooking, etc.), child (sex of child); and contextual (place of residence, region of residence) factors [2, 4, 5, 12–21]. Some previous studies in Bangladesh showed that maternal characteristics; child, and contextual factors were significantly associated with LBW [2, 9, 12, 22–25]. Additionally, a previous study conducted in Bangladesh identified that LBW was a significant factor in the likelihood of stunting and being underweight among under-five children [26, 27]. Most of those studies that previous carried out considered maternal perceptions of baby size at birth as proxy indicator for birth weight [19, 28, 29]. However, the current understanding of the determinants of LBW and its association with adverse health and nutritional outcomes has not been adequately studied using estimated weight of birth from more recent nationally representative sample of Bangladesh. Moreover, environmental factors other than household air pollution have not been broadly studied as potential risk factors for LBW in Bangladesh [30]. Hence, the current study aimed to identify various determinants, related to maternal factors, children characteristics, contextual factors and environmental factors, of LBW and further extended to determine the effect of low birthweight on adverse health and nutritional outcomes of children 0–23 months using a nationally representative cross-sectional survey.

## Methods

### Data and sampling

**Fig. 1:**
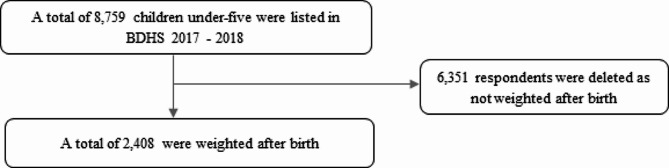
Sample size selection

A cross sectional nationally representative data from Bangladesh Demography and Health Survey (BDHS) 2017–2018 was used in this study. Demographic Health Survey (DHS) covers information regarding demographic and social factors as well as health and nutritional indicators for adults (both male and female) and children to monitor a wide range of the population. The BDHS 2017-18 was a multistage sampling. In the first stage, 675 primary sampling units (PSUs) were selected of which 250 PSUs were from urban and 425 PSUs were from rural areas. The PSUs were based on enumeration areas (clusters) listed in the population census 2011 conducted by the Bangladesh Bureau of Statistics. The second stage involved selecting an average of 30 households from each PSU using an equal probability systematic sampling technique. The multistage sampling and corresponding sampling weight might help to reduce potential sampling bias. In addition, all ever-married women aged 15–49 years (with or without children aged less than 5 years) from the preselected households were interviewed without replacement and change in the implementing stage to prevent selection bias. A total of 20,127 women aged 15–49 years were interviewed from 19,457 households with a response rate of 98.8% [31]. In BDHS 2017-18, a total of 8,759 children under-five were listed and birth weight was able to collect from a written record for 2,408 children aged 0–23 months of age (Fig. 1 and Additional file: Table S1).

### Major outcome variable

LBW, the child’s adverse health (e.g., fever, cough, acute respiratory infection (ARI), diarrhea), and child’s nutritional status (e.g., stunting, wasting and underweight) were considered outcome variables in this study. All outcome variables were coded as binary (1 for yes and 0 for no).

LBW: Child’s birth weight below 2.5 kg regardless of gestational age was considered LBW. If the child’s birth weight less than 2.5 kg coded as 1, otherwise coded as 0 [31].

### Other outcome variables

#### Child’s adverse health outcomes

Fever: Children who had a fever prior two weeks before the survey was categorized as 1; otherwise categorized as 0 [31].

Cough: Children who had a cough prior two weeks of the survey was categorized as 1; otherwise categorized as 0 [31].

ARI: Children had symptoms of ARI (short, rapid breathing which was chest-related, and/or difficult breathing which was chest-related) in the 2 weeks preceding the survey was categorized as 1; otherwise categorized as 0 [31].

Diarrhea: Children who had diarrhea in the 2 weeks preceding the survey was categorized as 1; otherwise categorized as 0 [31].

Had at least one illness: Children who had at least one of the conditions among fever, cough, ARI, and diarrhea in the 2 weeks preceding the survey was considered having at least one illness and categorized as 1; otherwise categorized as 0.

#### Child’s nutritional status

Stunting: A child was considered to be stunted (short stature for age), if the height-for-age, index was 2 standard deviations or more below the respective median of the World Health Organization reference population and was categorized as 1; otherwise categorized as 0 [32].

Wasting: A child was considered wasted (perilously thin) if the weight-for-height index was 2 standard deviations or more below the respective median of the World Health Organization reference population and was categorized as 1; otherwise categorized as 0 [32].

Underweight: A child was considered to be underweight (low weight for age) if the weight-for-age index was 2 standard deviations or more below the respective median of the World Health Organization reference population and was categorized as 1; otherwise categorized as 0 [32].

At least one undernutrition condition: Children who had at least one of the conditions among stunting, wasting, and/or underweight were considered having at least one undernutrition condition and was categorized as 1; otherwise categorized as 0.

### Independent variables

Various maternal and child characteristics and contextual and environmental factors found significant in previous literature and/or available in BDHS 2017-18 dataset were used as independent variables in this study [2, 4, 5, 12–20]. Maternal factors included mother’s age in years (15–19, 20–24, 25–29, 30–34, 35 and above); parents’ educational status (both parents were uneducated, only father was uneducated, only mother was uneducated, both parents were educated); mother currently working (no, yes); underweight mother (no, yes); mothers’ decision-making autonomy (not practiced, practiced); mother’s attitudes towards violence (not justified, justified); mothers received antenatal care (ANC) (no, yes); the number of living children (≤ 2, ≥ 3); age at first sex in years (< 15, 15–24, 25–34); wanted last child (wanted then, wanted later, wanted no more); ever had terminated pregnancy (no, yes); last birth with a caesarean section (no, yes); and a sign of pregnancy complication (no, yes). Sex of child (male, female) was listed as child characteristics. Contextual factors included mass media exposure (no, yes); wealth index (poorest, poorer, middle, richer, richest), and place of residence (urban, rural). Home environmental factors were types of drinking water (improved, unimproved); type of toilet facility (improved, unimproved); solid waste used for cooking (nonsolid, solid)( Additional file: Table S1).

### Statistical analysis

Descriptive statistics were used to evaluate the background characteristics of the respondents. A Chi-square test was used to find out the association between outcome and independent variables. The statistical significance was set at *p* < 0.25 (two-tailed), rather than the typical cut-off point of 0.05, which may aid to include the factors that are considered to be important [33]. Multivariable logistic regression analyses was used to find out the effects of independent variables on outcome measures. Factors found significant in the Chi-square test were simultaneously entered into the Multivariable logistic regression model. In this study, factors significantly associated with LBW were identified using multivariable logistic regression analysis. Further, multivariable logistic regression analysis was used to find out the effect of LBW on adverse nutritional and health outcomes. The magnitude of the association was assessed using adjusted odds ratio (AOR) and confidence interval (CI) in multivariable logistic regression. The significance level for multivariable logistic regression analyses was set at *p* < 0.05 (two-tailed). Multicollinearity was checked by examining the standard errors (SEs) of regression coefficients in the logistic regression analyses. An SE > 2.0 indicates multicollinearity among the independent variables [34]. The SEs for the independent variables in the adjusted models for each outcome were < 1, indicating an absence of multicollinearity. Akaike information criterion (AIC), and Bayesian information criterion (BIC) were assessed for model’s evaluation. Stata version 14.2 (StataCorp LP, College Station, Texas) was used for all analyses. To adjust the complex nature of the sampling, such as, sampling weight, cluster, and strata; the Stata command ‘svyset’ was prepared and used.

## Results

### Background characteristics

More than one-third (36.6%) of all mothers belong to the age group 20–24 years. Only 2.9% of mothers of children were uneducated (only mother 1.6% and both parents 1.3%). Approximately one out of ten mothers (12.1%) was underweight, and 98% of mothers received antenatal care. About two-thirds (65.5%) of the total mothers were rural dwellers, and only 10.9% were from the poorest section the society. Around 38.1% of children living in households had unimproved toilet facilities and 59.2% of children in households used solid waste for cooking. The detailed background characteristics are presented elsewhere (Table 1).

**Table 1:** Background characteristics of the respondents (weighted frequency)

Factors	Number	Frequency
**Maternal factors**		
**Mother’s age (in years)**		
15–19	445	18.5
20–24	882	36.6
25–29	604	25.1
30–34	354	14.7
35 and over	123	5.1
**Parents’ education**		
Both parents were uneducated	30	1.3
Only father was uneducated	148	6.2
Only mother was uneducated	38	1.6
Both parents were educated	2168	90.9
**Mother currently working**		
No	1656	68.8
Yes	752	31.2
**Underweight mother**		
No	2117	87.9
Yes (< 18.5 kg/m^2^)	291	12.1
**Mothers’ decision-making autonomy**		
Not practiced	339	14.2
Practiced	2046	85.8
**Mother’s attitudes towards violence**		
Not justified	2030	84.3
Justified	378	15.7
**Mothers received antenatal care**		
No	43	1.9
Yes	2261	98.1
**Number of living children**		
≤ 2	1997	82.9
≥ 3	411	17.1
**Age at first sex (in years)**		
< 15	381	15.8
15–24	1949	80.9
25–34	78	3.2
**Wanted last child**		
Wanted then	1968	81.8
Wanted later	307	12.7
Wanted no more	133	5.5
**Ever had terminated pregnancy**		
No	2002	83.1
Yes	406	16.9
**Last birth a caesarean section**		
No	894	37.2
Yes	1507	62.8
**Sign of Pregnancy complication**		
No	1310	58.0
Yes	951	42.1
**Child characteristics**		
**Sex of child**		
Male	1291	53.6
Female	1117	46.4
**Low birth weight**		
No	2,016	83.7
Yes	392	16.3
**Contextual factors**		
**Mass media exposure**		
No	512	21.3
Yes	1896	78.8
**Wealth index**		
Poorest	262	10.9
Poorer	354	14.7
Middle	458	19.0
Richer	570	23.7
Richest	764	31.7
**Place of residence**		
Urban	831	34.5
Rural	1577	65.5
**Environmental factors**		
**Type of drinking water**		
Improved	2020	83.9
Unimproved	388	16.1
**Type of toilet facility**		
Improved	1490	61.9
Unimproved	918	38.1
**Solid waste used in cooking**		
No	981	40.8
Yes	1424	59.2
**Total**	**2408**	**100.0**

**Fig. 2:**
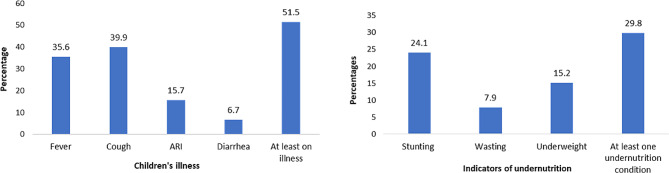
Children’s adverse health and nutritional outcomes (0–23 months of age)

According to Fig. 1, around 15.7% and 6.7% of children aged 0–23 months suffered from ARI and diarrhea respectively. More than half of the children (51.5%) had at least one illness among fever, cough, ARI and Diarrhea. Around 24.1% of children had stunting, 7.9% had wasting and 15.2% were underweight. Around 29.8% of children had at least one under-nutritional condition ( Fig. 2).

### Prevalence and determinants of LBW

The prevalence of LBW was significantly higher among children of mothers with no formal education (fathers were educated) (41.5%), children from the poorest socioeconomic status (22.4%), mothers who had more than 3 living children (18.8%), wanted child later (19.8%), and children were born by normal delivery (18.6%) (Table 2). The prevalence of LBW was significantly higher in household with unimproved toilet facilities (18.3%) (Table 2).

**Table 2:** Prevalence and determinants of LBW

Factors	Prevalence of LBW			Determinants	
	Number	Prevalence (95% CI)	*p* values	AOR (95% CI)	*p* values
**Mother’s age (in years)**					
15–19	70	17.2 (13.4, 21.8)	0.863		
20–24	139	16.7 (13.9, 19.8)			
25–29	91	14.6 (11.5, 18.3)			
30–34	56	16.8 (12.3, 22.5)			
35 and over	25	16.9 (11.1, 24.9)			
**Parents’ education**					
Both parents were uneducated	5	21.8 (9.0, 44.0)	0.001	1.35 (0.55–3.30)	0.517
Only father was uneducated	27	20.4 (13.8, 29.1)		1.13 (0.71–1.81)	0.597
Only mother was uneducated	17	41.5 (26.1, 58.8)		2.64 (1.27–5.50)	0.010
Both parents were educated	330	15.6 (13.8, 17.5)		1.00	
**Mother currently working**					
No	265	16.6 (14.5, 19.0)	0.589		
Yes	116	15.6 (12.9, 18.7)			
**Underweight mother**					
No	332	16.3 (14.5, 18.3)	0.892		
Yes (BMI < 18.5 kg/m^2^)	49	16.0 (11.9, 21.1)			
**Mothers’ decision-making autonomy**					
Not practiced	50	13.9 (10.2, 18.5)	0.227		
Practiced	329	16.8 (14.9, 18.8)			
**Mother’s attitudes towards violence**					
Not justified	326	16.4 (14.5, 18.5)	0.818		
Justified	55	15.8 (12.2, 20.3)			
**Mothers received antenatal care**					
No	5	13.1 (5.2, 29.6)	0.711		
Yes	337	15.5 (13.9, 17.3)			
**Number of living children**					
≤ 2	300	15.7 (14.0, 17.7)	0.163	1.00	
≥ 3	81	18.8 (14.8, 23.7)		1.16 (0.82–1.63)	0.399
**Age at first sex (in years)**					
< 15	59	15.9 (12.3, 20.4)	0.633		
15–24	313	16.5 (14.6, 18.6)			
25–34	9	11.8 (5.4, 24.0)			
**Wanted last child**					
Wanted then	301	15.9 (14.1, 17.9)	0.191	1.00	
Wanted later	60	19.8 (15.2, 25.5)		1.31 (0.94–1.82)	0.109
Wanted no more	20	13.2 (8.2, 20.7)		0.75 (0.42–1.36)	0.345
**Ever had terminated pregnancy**					
No	313	16.1 (14.3, 18.1)	0.711		
Yes	68	16.9 (13.2, 21.4)			
**Last birth a caesarean section**					
No	161	18.6 (15.8, 21.9)	0.035	1.00	
Yes	219	14.9 (12.9, 17.1)		0.85 (0.66–1.08)	0.179
**Sign of pregnancy complication**					
No	204	16.8 (14.5, 19.3)	0.064	1.00	
Yes	133	13.7 (11.5, 16.1)		0.82 (0.64–1.04)	0.094
**Sex of child**					
Male	190	14.8 (12.7, 17.1)	0.056	1.00	
Female	191	18.0 (15.5, 20.8)		1.31 (1.04–1.65)	0.023
**Mass media exposure**					
No	86	17.5 (14.0, 21.6)	0.466		
Yes	295	15.9 (14.0, 18.1)			
**Wealth index**					
Poorest	55	22.4 (17.1, 28.8)	0.087	1.69 (1.13–2.51)	0.010
Poorer	49	15.6 (11.4, 21.0)		1.16 (0.79–1.70)	0.461
Middle	79	18.1 (14.5, 22.4)		1.41 (1.01–1.98)	0.046
Richer	85	15.4 (12.1, 19.3)		1.17 (0.84–1.62)	0.356
Richest	113	14.1 (11.3, 17.4)		1.00	
**Place of residence**					
Urban	166	16.5 (13.9, 19.4)	0.863		
Rural	215	16.2 (14.0, 18.6)			
**Type of drinking water**					
Improved	320	16.4 (14.5, 18.5)	0.805		
Unimproved	61	15.7 (11.8, 20.7)			
**Type of toilet facility**					
Improved	222	15.0 (12.9, 17.4)	0.078	1.00	
Unimproved	159	18.3 (15.6, 21.5)		1.38 (1.03–1.84)	0.030
**Solid waste used in cooking**					
No	146	15.1 (12.5, 18.1)	0.363		
Yes	233	16.9 (14.6, 19.4)			
**Total**	381	16.3 (14.6, 18.1)			
**AIC**	1921.2				
**BIC**	2018.1				

AIC, Akaike information criterion; AOR, adjusted odds ratio; BIC, Bayesian information criterion; CI, Confidence interval

From regression analysis results, mothers with no formal education (fathers were educated), female children, and children from the poorest socio-economic status had significant effect on the LBW. Children were 2.64 times (AOR = 2.64, 95% CI = 0.55–3.30, *p* = 0.010) more likely to born with LBW among mothers with no formal education than educated mothers. Female children had 1.3 times (AOR = 1.31, 95% CI = 1.04–1.65, *p* = 0.023) higher chances of being LBW than their counterparts. Children from the poorest socioeconomic background (AOR = 1.69, 95% CI = 1.13–2.51, *p* = 0.010) were more likely to born LBW than the children from the richest socio-economic status. Similarly, the likelihood of being LBW at birth was 1.26 times (AOR = 1.34, 95% CI = 1.03–1.84, *p* = 0.030) higher among children living in household with unimproved toilet facilities (Table 2). The unadjusted regression models were presented in Table S2 (Additional file: Table S2).

### Effects of LBW on adverse health and nutritional status

The prevalence of LBW was significantly higher among children who had at least one under-nutritional condition (48.1%), had stunting (40%), being underweight (30.4%), and wasting (10.7%) (Table 3).

**Table 3:** Effects of LBW on child’s adverse health and nutritional status

Outcomes	Exposure	Prevalence			Determinants	
		Number	Prevalence (95% CI)	*p* values (χ^2^)	AOR (95% CI)	*p* values
**Children’s adverse health status**						
**Fever**	**Low birth weight**					
	No	688	34.9 (32.6, 37.3)		1.00	
	Yes	137	39.6 (33.6, 46.0)	0.150	1.26 (1.02–1.60)	0.047
**Cough**	**Low birth weight**					
	No	794	40.4 (37.8, 43.0)			
	Yes	133	37.0 (31.2, 43.1)	0.311		
**ARI**	**Low birth weight**					
	No	286	15.2 (13.4, 17.1)		1.00	
	Yes	59	18.6 (14.4, 23.6)	0.156	1.25 (0.92–1.71)	0.144
**Diarrhea**	**Low birth weight**					
	No	140	6.6 (5.5,8.0)			
	Yes	28	7.3 (4.8, 10.8)	0.689		
**Had at least one illness**	**Low birth weight**					
	No	1017	51.6 (49.0, 54.1)			
	Yes	182	51.1 (44.5, 57.7)	0.898		
**Children’s adverse health status**						
**Stunting**	**Low birth weight**					
	No	391	21.2 (19.2, 23.4)		1.00	
	Yes	140	40.0 (34.1, 46.2)	< 0.001	2.42 (1.86–3.15)	< 0.001
**Wasting**	**Low birth weight**					
	No	132	7.3 (5.9, 8.9)		1.00	
	Yes	34	10.7 (7.4, 15.3)	0.047	1.49 (1.02–2.25)	0.049
**Underweight**	**Low birth weight**					
	No	244	12.4 (10.7, 14.3)		1.00	
	Yes	112	30.4 (25.4, 35.9)	< 0.001	3.19 (2.40–4.23)	< 0.001
**At least one under-nutrition condition**	**Low birth weight**					
	No	520	28.2 (25.8, 30.8)		1.00	
	Yes	165	48.1 (42.1, 54.2)	< 0.001	2.36 (1.83–3.03)	< 0.001

AOR, adjusted odds ratio, ARI, acute respiratory infections, CI, Confidence interval

For each outcome, model was adjusted for children’s age, children’s sex, parental educational status, wealth index and place of residence

Furthermore, LBW had significant effect on children who had a fever, with stunting, wasting, being underweight and with at least one under-nutritional condition (see Table 2). Children who were LBW had 1.26 times (AOR = 1.26, 95% CI = 1.02–1.60, *p* = 0.047) higher chance of getting fever than a normal child. Children born with LBW were 2.4 times (AOR = 2.42, 95% CI = 1.86–3.15, *p* < 0.001); 3 times (AOR = 3.19, 95% CI = 2.40–4.23, *p* < 0.001) and 1.49 times (AOR = 1.49, 95% CI = 1.02–2.25, *p* = 0.049) respectively, more likely of being stunted, wasted and underweight than normal children. Similarly, LBW had a significant effect on children with at least one undernutrition condition (AOR = 2.39, 95% CI = 1.83–3.03, *p* < 0.001) (Table 3). The unadjusted regression models were presented in Table S3 (Additional file: Table S3).

## Discussion

The current study extensively assessed determinants of LBW and identified its effect on adverse health and nutritional outcome of children using a nationally represented sample in Bangladesh. This study found that prevalence of LBW in Bangladesh stood at 16.3%, similar to rates in neighboring countries like India with 16.4% and Pakistan with 16.9% [10, 14, 35, 36]. The prevalence of LBW was slightly lower in Nepal, and Sri Lanka which accounted for 15.4%, and 14.6%, respectively [14, 37]. Countries in South Asia exhibited comparable patterns of prevalence for LBW; it is perhaps due to similarities between countries in terms of geography, culture, economy, and quality of life [38].

The study showed that the prevalence of LBW was higher among children of mothers with no formal education and children from the poorest socio-economic status. Additionally, children of mothers with no formal education, being a female child and children from the poorest socio-economic status were more likely of being LBW. In previous literatures, mother’s education, child sex and wealth index were found significant factors of LBW in Bangladesh [2, 23–25]. Findings of the present study were consistent with previous studies conducted in other neighboring countries [5, 10, 14, 39]. Mothers who belonged to poor socio-economic background may also lack an educational profile, often experience difficulties in accessing nutrition and health care, which can result in inadequate maternal nutrition during pregnancy leading to maternal undernutrition and consequently LBW [13, 40]. Lack of education can also limit access to prenatal care, which might hinder the mother’s ability to receive proper medical care [21, 41]. Although Bangladesh has gained substantial improvement in female education over the past few decades, unfortunately approximately 36% of females still remain illiterate [42]. Female dropout rates were very high including 13.3% in primary and 40.29% in secondary school level [43]. The government of Bangladesh has taken initiatives such as stipends, allowances, and free education facilities to reduce the female dropout rate at school. Still, it needs to strengthen administrative coordination, establish a monitoring and evaluation framework, and increase multidimensional investment in education to improve female education and consequently health status. Furthermore, no preference for female children is often responsible for poor ANC visits and inadequate nutritional practice among mothers during pregnancy results in adverse birth outcome like LBW [44]. Despite substantial progress in primary health care over the last decades, only 47% of pregnant women in Bangladesh receive at least four ANC visits [45]. A lack of access to health providers and facilities has contributed to nearly one in two mothers in Bangladesh not receiving four or more ANC visits from skilled health professionals [46]. In addition, gender inequality, cultural and religious behavior and restrictions among women; illiteracy and poverty are often considered the preference of male child as well as poor ANC visit in Bangladesh [47]. Improving access to quality ANC and sustaining its implementation must be prioritized for the country to achieve better health sustainability.

The study also revealed that the odds of being LBW was higher in household with unimproved toilet facility as well as it was estimated higher prevalence of LBW in those households in Bangladesh. Recent studies conducted in Bangladesh did not find any correlation between the type of toilet facilities and LBW [23]. Open defecation and unsafe bowel disposal negatively affect the nutrition and health status of pregnant women and promote chronic infections [48]. Due to unimproved toilet facilities, especially in urban slums and rural areas, women also suffer from diarrhea and hookworm infestation which lead to maternal anemia, undernutrition, and infectious diseases that results in poor pregnancy outcomes like LBW [8, 10, 13, 48, 49]. Sufficient budget allocation and ensuring effective implementation of resources under national sanitation program can provide a framework for addressing sanitation issues and improving access to clean water and hygienic toilet facilities. In addition, promoting good sanitation practices and increasing awareness about the importance of sanitation and hygiene can help prevent the spread of disease and improve maternal and child health outcomes.

the children with LBW were more likely to suffer from fever and undernutrition than normal children. Previous studies based on data from Bangladesh showed that LBW was identified as an important risk factor for various forms of undernutrition [26, 27]. Several neighboring countries like India, and Pakistan found comparable results [50, 51]. Another study in Africa (Malawi) also found higher odds of stunting, wasting, and being underweight among LBW children [52]. LBW infants often had difficulties in feeding due to underdeveloped digestive systems or a weak sucking reflex, which can lead to inadequate intake of nutrients [50, 53]. Moreover, LBW infants may have higher metabolic rates, which means they require more energy and nutrients per kilogram of body weight than a normal infant and this supply-demand imbalance leads to undernourishment [54]. The children of LBW had lower immune substances and improper formation of the respiratory tract which lead to various infectious diseases like pneumonia and often suffer from fever, and cough [55, 56]. LBW children and their mothers need adequate parenteral care and nutritional education including regular checkup and nutritional counselling, initiation of early and exclusive breast feeding, and nutrient-dense complementary foods to reduce the incidence of child morbidity and undernutrition in children born with LBW.

The main strength of this study was the utilization of nationally representative cross-sectional sample which covers both rural and urban areas of all districts of the country as well as aids to generalize the findings. Additionally, BDHS 2017–2018 data was collected by using a standard questionnaire, designing a complex survey strategy, and global study model to provide credible results. Despite these advantages, we acknowledged several limitations of this study. As the data was collected based on the mother’s self-reported information, the information might be affected by recall bias. This differential misclassification could cause either an overestimation or underestimation of the study findings. The cross-sectional nature of the data interferes with drawing causal associations between dependent and independent variables. This study might limit to generalize the findings only for low- and middle- income countries.

## Conclusion

One out of five children were born with LBW in Bangladesh. Poor maternal education, female child, poorest socio-economic status, and unimproved toilet facilities were significantly associated with LBW. Further, the likelihood of gaining illness and being undernutrition was higher in LBW children. To improve maternal pregnancy, and child health outcomes, it is crucial to implement policies that tackle poverty, gender inequality, and social disparities. Encouraging regular antenatal care visits and early medical intervention is essential, as is promoting education and awareness about reproductive health, hygiene and safe sanitation practices. Further, treating a low birth weight (LBW) child to reduce adverse health and nutritional outcomes needs child malnutrition multifaceted approach including exclusive breastfeeding promotion, nutritional intervention, growth monitoring, accessible medical care, and education of caregivers.

### Electronic supplementary material

Below is the link to the electronic supplementary material.

Supplementary Material 1

Supplementary Material 2

Supplementary Material 3

Supplementary Material 4

## Data Availability

The BDHS 2017-18 data is publicly available on the DHS Program’s page at https://dhsprogram.com/data/.

## Abbreviations

ANC: Antenatal care
AOR: adjusted odds ratio
ARI: Acute respiratory infection
BDHS: Bangladesh Demography and Health Survey
CI: Confidence interval
LBW: Low birth weight
PSU: Primary sampling unit
