# Supplementary material for: Determinants of low birth weight and its effect on childhood health and nutritional outcomes in Bangladesh
Source: J Health Popul Nutr. 2024 May 13;43:64. doi: 10.1186/s41043-024-00565-9 (PMC11092222; doi:10.1186/s41043-024-00565-9)
Supplement: Supplementary file 2 — Supplementary Material 2 [file 41043_2024_565_MOESM2_ESM.docx]

**Determinants of low birth weight and its effect on childhood health and nutritional outcomes in Bangladesh. Do home environmental factors matter?**

Md. Zahidul Islam^1^^¶^, BPH; Mohammad Rocky Khan Chowdhury^1,2¶^, MSc, MPhil; Manzur Kader^3^, MSc, PhD; Baki Billah^2^, MSc, PhD; Md. Shariful Islam^1^, MSc, MPhil; Mamunur Rashid^4^, MMSc, PhD

^1^Department of Public Health, First Capital University of Bangladesh, Chuadanga-7200, Bangladesh

^2^Department of Epidemiology and Preventive Medicine, School of Public Health and Preventive Medicine, Monash University, Melbourne, Australia

^3^Department of Medicine Solna, Clinical Epidemiology Division, Karolinska Institutet, Maria Aspmans Gata 30A, 17176 Stockholm, Sweden.

^4^Department of Public Health and Sports Sciences, University of Gävle, Sweden

¶ Md. Zahidul Islam and Mohammad Rocky Khan Chowdhury have equal contribution


**Article summary**

Abstract word count: 293
Manuscript word count: 3,196

No of tables: 4

No of figures: 1

No of references: 49

**Corresponding Author**

Mamunur Rashid
