# Supplementary material for: Determinants of low birth weight and its effect on childhood health and nutritional outcomes in Bangladesh
Source: J Health Popul Nutr. 2024 May 13;43:64. doi: 10.1186/s41043-024-00565-9 (PMC11092222; doi:10.1186/s41043-024-00565-9)
Supplement: Supplementary file 3 — Supplementary Material 3 [file 41043_2024_565_MOESM3_ESM.docx]

**Table S1** Definition of independent variables used in the study

| **Variable** | **Description** | **Measurement** | **Scale of measurement** | **No of response /Missing** |
| --- | --- | --- | --- | --- |
| Low birth weight (Outcome variable) | When a baby is born weighing less than 2500 grams (2.5 kg) | No, Yes | Binary | 2,408/0 |
| **Maternal characteristics** | | | | |
| Mothers’ age (in years) | Age of mothers at the time of data collection | 15-19, 20-24, 25-29, 30-34, 35 and above | Discrete, categorical | 2,408/0 |
| Parents’ educational status | Parental educational status. Accomplishment of at least five years of schooling (primary level, class 1 to 5) refers educated. No (0 years of schooling) schooling year refers uneducated. | Both parents were uneducated, only father was uneducated, only mother was uneducated, both parents were educated | Categorical | 2,408/0 |
| Mother currently working | Mother engaged in economic activity at the time of data collection. | No, Yes | Binary | 2,408/0 |
| Underweight mother | Mother with <18.5 kg/m^2^ of body mass index refers to underweight, and ≥18.5 kg/m^2^ refers to healthy or not underweight | No,  Yes (<18.5kg/m^2^) | Binary | 2,408/0 |
| Mothers’ decision-making autonomy | In the BDHS surveys, a woman’s decision-making power is assessed on the following three themes: 1) a woman who usually decides on her healthcare 2) a woman who usually decides on large household purchases and 3) a woman who usually decides on visits to family or relatives. The response options were as follows: (a) respondent alone, (b) respondent and husband/partner, (c) respondent and another person, (d) husband/partner alone, (e) someone else, (f) other. For each question, a value of 1 was assigned for inability in decision-making if the responses were d, e, or f and 0 for otherwise if the responses were a, b, or c. The values were then added, resulting in a score from 0 to 3. The Cronbach’s α for the instruments was 0·79, indicating high internal consistency. | Not participated, Participated | Binary | 2,408/0 |
| Mother’s attitudes toward violence | Respondents are asked if they agree that a husband is justified in hitting or beating his wife under each of the following five circumstances: she burns the food, she argues with him, she goes out without telling him, she neglects the children, and she refuses to have sex with him. If respondents answer “yes” in at least one circumstance, they are considered to have attitudes justifying wife beating. | Not justified, Justified | Binary | 2,408/0 |
| Mothers received antenatal care | At least one medical surveillance and review performed during pregnancy for the early detection of possible complications of pregnancy. | No, Yes | Binary | 2,408/0 |
| Number of living children | The number of alive children to mothers at the time of data collection. | ≤ 2, ≥ 3 | Discrete, categorical | 2,408/0 |
| Age at first sex (in years) | Age at first sexual intercourse told by mother. | < 15, 15-24, 25-34 | Discrete, categorical | 2,408/0 |
| Wanted last child | The child was unexpected or expected at the time of birth | Wanted then, wanted later, wanted no more. | Categorical | 2,408/0 |
| Ever had terminated pregnancy | Women who ever had a pregnancy that was miscarried, aborted, or ended in a stillbirth. | No, Yes | Binary | 2,408/0 |
| Last birth a caesarean section | Women who delivered their last child through caesarean section. | No, Yes | Binary | 2,408/0 |
| Sign of Pregnancy complication | The most common danger signs of pregnancy complications were vaginal bleeding, severe headache, convulsions or fits, high fever, abdominal pain, vision problem, absence of foetal movements, gush of fluid from vagina, and foul-smelling vaginal discharge. The presence of any one of these signs was considered as “Yes”. | No, Yes | Binary | 2,408/0 |
| **Children’s characteristics** | | | | |
| Sex of child | Sex differential of children | Male, Female | Binary | 2,408/0 |
| **Contextual factors** | | | | |
| Mass media exposure | Mass media exposure through television, radio and newspaper/magazine has been defined as exposure to at least one media that exposes to at least once a week | No, Yes | Binary | 2,408/0 |
| Wealth index | Wealth index in the DHS surveys is calculated, by the DHS authority, based on information on household characteristics and assets using principal component analysis. Then households are classified into quintiles based on the values of the wealth index, where households with lower values of the index is considered as poorest and vice-versa | Poorest, poorer, middle, richer, richest | Categorical | 2,408/0 |
| Place of residence | Different residential areas across the country. | Urban, rural | Binary |  |
| **Home environmental factors** | | | | |
| Types of drinking water | Improved: piped into dwelling, piped to yard/plot, public tap/standpipe, piped to neighbour, tube well or borehole, protected well, protected spring, rainwater, tanker truck, cart with small tank, bottled water; unimproved: unprotected well, unprotected spring, surface water (river, dam, lake, pond, stream, canal, irrigation channel), other | Improved, unimproved | Binary | 2,408/0 |
| Type of toilet facility | Improved: flush - to piped sewer system, flush - to septic tank, flush - to pit latrine, flush - don't know where, pit latrine - ventilated improved pit, pit latrine - with slab, composting toilet; unimproved: flush - to somewhere else, pit latrine - without slab / open pit, bucket toilet, hanging toilet/latrine, other | Improved, unimproved | Binary | 2,408/0 |
| Solid waste used for cooking | Use of solid fuel includes coal, lignite, charcoal, wood, straw/shrubs/grass, agricultural crop and animal dung considered as “solid”. | Nonsolid, Solid | Binary | 2,408/0 |

**Table S2** Prevalence and determinants of LBW (results of simple logistic regression)

| **Factors** | **Determinants** | |
| --- | --- | --- |
|  | **AOR (95% CI)** | ***p* values** |
| **Mother’s age (in years)** |  |  |
| 15-19 | 1.00 |  |
| 20-24 | 0.93 (0.68-1.28) | 0.674 |
| 25-29 | 0.86 (0.61-1.21) | 0.403 |
| 30-34 | 0.95 (0.65-1.40) | 0.812 |
| 35 and over | 1.11 (0.67-1.84) | 0.677 |
| **Parents’ education** |  |  |
| Both parents were uneducated | 1.37 (0.51-3.69) | 0.526 |
| Only father was uneducated | 1.22 (0.80-1.89) | 0.353 |
| Only mother was uneducated | 4.06 (2.14-7.70) | <0.001 |
| Both parents were educated | 1.00 |  |
| **Mother currently working** |  |  |
| No | 1.00 |  |
| Yes | 0.93 (0.73-1.17) | 0.521 |
| **Underweight mother** |  |  |
| No | 1.00 |  |
| Yes (BMI <18.5 kg/m^2^) | 1.06 (0.77-1.48) | 0.714 |
| **Mothers’ decision-making autonomy** |  |  |
| Not practiced | 0.94 (0.68-1.30) | 0.719 |
| Practiced | 1.00 |  |
| **Mother’s attitudes towards violence** |  |  |
| Not justified | 1.00 |  |
| Justified | 0.99 (0.73-1.35) | 0.960 |
| **Mothers received antenatal care** |  |  |
| No | 0.98 (0.38-2.54) | 0.961 |
| Yes | 1.00 |  |
| **Number of living children** |  |  |
| ≤ 2 | 1.00 |  |
| ≥ 3 | 1.36 (1.04-1.79) | 0.024 |
| **Wanted last child** |  |  |
| Wanted then | 1.00 |  |
| Wanted later | 1.31 (0.96-1.77) | 0.087 |
| Wanted no more | 0.97 (0.59-1.59) | 0.913 |
| **Ever had terminated pregnancy** |  |  |
| No | 1.00 |  |
| Yes | 1.03 (0.78-1.38) | 0.808 |
| **Last birth a caesarean section** |  |  |
| No | 1.00 |  |
| Yes | 0.77 (0.62-0.97) | 0.024 |
| **Sign of pregnancy complication** |  |  |
| No | 1.00 |  |
| Yes | 0.80 (0.64-1.01) | 0.071 |
| **Sex of child** |  |  |
| Male | 1.00 |  |
| Female | 1.21 (0.97-1.51) | 0.087 |
| **Mass media exposure** |  |  |
| No | 1.05 (0.81-1.37) | 0.682 |
| Yes | 1.00 |  |
| **Wealth index** |  |  |
| Poorest | 1.65 (1.15-2.36) | 0.006 |
| Poorer | 1.02 (0.72-1.48) | 0.880 |
| Middle | 1.37 (1.00-1.88) | 0.048 |
| Richer | 1.11 (0.82-1.51) | 0.489 |
| Richest | 1.00 |  |
| **Place of residence** |  |  |
| Urban | 1.00 |  |
| Rural | 1.00 (0.80-1.25) | 0.975 |
| **Type of drinking water** |  |  |
| Improved | 1.00 |  |
| Unimproved | 1.02 (0.76-1.38) | 0.892 |
| **Type of toilet facility** |  |  |
| Improved | 1.00 |  |
| Unimproved | 1.38 (1.08-1.87) | 0.036 |
| **Solid waste used in cooking** |  |  |
| No | 1.00 |  |
| Yes | 1.12 (0.89-1.40) | 0.329 |

AOR, Adjusted odds ratio, CI, Confidence interval

**Table S3** Associations between low birth weight and child’s adverse health and nutritional outcomes (results of simple logistic regression)

| **Outcomes** | **Exposure** | **Determinants** | |
| --- | --- | --- | --- |
|  |  | **AOR (95% CI)** | ***p* values** |
| **Fever** | **Low birth weight** |  |  |
|  | No | 1.00 |  |
|  | Yes | 1.17 (0.93-1.48) | 0.177 |
| **Cough** | **Low birth weight** |  |  |
|  | No | 1.00 |  |
|  | Yes | 0.89 (0.70-1.12) | 0.325 |
| **ARI** | **Low birth weight** |  |  |
|  | No | 1.00 |  |
|  | Yes | 1.17 (0.87-1.60) | 0.300 |
| **Diarrhea** | **Low birth weight** |  |  |
|  | No | 1.00 |  |
|  | Yes | 1.12 (0.73-1.71) | 0.596 |
| **Had at least one illness** | **Low birth weight** |  |  |
|  | No | 1.00 |  |
|  | Yes | 0.99 (0.79-1.24) | 0.924 |
| **Stunting** | **Low birth weight** |  |  |
|  | No | 1.00 |  |
|  | Yes | 2.39 (1.89-3.02) | <0.001 |
| **Wasting** | **Low birth weight** |  |  |
|  | No | 1.00 |  |
|  | Yes | 1.39 (0.94-2.06) | 0.103 |
| **Underweight** | **Low birth weight** |  |  |
|  | No | 1.00 |  |
|  | Yes | 3.00 (2.31-3.88) | <0.001 |
| **At least one under-nutrition condition** | **Low birth weight** |  |  |
|  | No | 1.00 |  |
|  | Yes | 2.24 (1.79-2.80) | <0.001 |

AOR, Adjusted odds ratio, ARI, acute respiratory infections, CI, Confidence interval
